# Maternal Intake of n-3 Polyunsaturated Fatty Acids During Pregnancy Is Associated With Differential Methylation Profiles in Cord Blood White Cells

**DOI:** 10.3389/fgene.2019.01050

**Published:** 2019-10-25

**Authors:** Marzia Bianchi, Anna Alisi, Marta Fabrizi, Cristina Vallone, Lucilla Ravà, Riccardo Giannico, Pamela Vernocchi, Fabrizio Signore, Melania Manco

**Affiliations:** ^1^Research Unit for Multifactorial Diseases, Bambino Gesù Children’s Hospital, IRCCS, Rome, Italy; ^2^Research Unit of Molecular Genetics of Complex Phenotypes, Bambino Gesù Children’s Hospital, IRCCS, Rome, Italy; ^3^Department of Obstetrics and Gynecology, Misericordia Hospital, Grosseto, Italy; ^4^Clinical Epidemiology Unit, Bambino Gesù Children’s Hospital, IRCCS, Rome, Italy; ^5^Unit of Human Microbiome, Bambino Gesù Children’s Hospital, IRCCS, Rome, Italy

**Keywords:** epigenetics, inflammation, insulin resistance, metabolic programming, pregnancy, polyunsaturated fatty acids

## Abstract

A healthy diet during pregnancy is pivotal for the offspring health at birth and later in life. N-3 polyunsaturated fatty acids (n-3 PUFAs) are not endogenously produced in humans and are exclusively derived from the diet. They are pivotal for the fetus growth and neuronal development and seem beneficial in reducing the risk of cardiometabolic diseases and preventing later allergic disorders in the offspring by modulating the inflammatory immune response. In the present study, we investigated the association between maternal intakes of n-3PUFAs, profiled on maternal erythrocyte membranes at pregnancy term, and offspring DNA methylation on cord blood mononuclear cells in a sample of 118 mother–newborn pairs randomly drawn from the “Feeding fetus’ low-grade inflammation and insulin-resistance” study cohort. N-3 PUFA content on erythrocyte membranes is a validated biomarker to measure objectively medium term intake of n-3 PUFAs. Based on distribution of n-3 PUFA in the whole cohort of mothers, we identified mothers with low (n-3 PUFA concentration <25th percentile), medium (n-3 PUFAs between 25th and 75th percentiles), and high n-3 PUFA content (>75th percentile). The HumanMethylation450 BeadChip (Illumina) was used for the epigenome-wide association study using the Infinium Methylation Assay. The overall DNA methylation level was not different between the three groups while there was significant difference in methylation levels at certain sites. Indeed, 8,503 sites had significantly different methylations between low and high n-3 PUFA groups, 12,716 between low and medium n-3 PUFA groups, and 18,148 between high and medium n-3 PUFA groups. We found differentially methylated genes that belong prevalently to pathways of signal transduction, metabolism, downstream signaling of G protein-coupled receptors, and gene expression. Within these pathways, we identified four differentially methylated genes, namely, MSTN, IFNA13, ATP8B3, and GABBR2, that are involved in the onset of insulin resistance and adiposity, innate immune response, phospholipid translocation across cell membranes, and mechanisms of addiction to high fat diet, alcohol, and sweet taste. In conclusion, findings of this preliminary investigation suggest that maternal intake of n-3 PUFAs during pregnancy has potential to influence the offspring DNA methylation. Validation of results in a larger cohort and investigation of biological significance and impact on the phenotype are warranted.

## Introduction

Omega-3 polyunsaturated fatty acids (n-3 PUFAs) are not endogenously produced in humans but derived from diet. They play a pivotal role in the human health from conception through every stage of human development, maturation, and ageing. N-3 PUFAs are essential for neurodevelopment and modulation of inflammatory immune response, being protective against allergic disorders (De Giuseppe et al., 2014; Schindler et al., 2016).

Several lines of evidence suggest that individuals with a diet rich in n-3 PUFAs have lower incidence of cardiovascular disease and, in general, of chronic non-communicable diseases (Li, 2015; Abdelhamid et al., 2018).

The mechanisms underlying the protective effect of n-3 PUFAs from the intrauterine life to the adulthood are incompletely understood but are thought to include altered eicosanoid metabolism and subsequent changes in cell signaling, transcription factor activity, and gene expression. Evidence in animal models and humans suggests that n-3 PUFAs influence global DNA methylation patterns at any stage of life (Burdge and Lillycrop, 2014).

In the womb, n-3 PUFA supply to the fetus depends strongly on maternal consumption and metabolism, as well as on efficiency of the placental transport (Hornstra, 2000; Kilari et al., 2009). In controlled investigations, supplementation during pregnancy with docosahexaenoic (DHA) and/or eicosopentanoic (EPA) acids was associated with changes in the offspring methylation levels of various genes, including genes coding for inflammatory mediators (Lee et al., 2013; Amarasekera et al., 2014; Lee et al., 2014; Van Dijk et al., 2016). Animal and human studies demonstrated that the *in utero* environment shapes the offspring epigenome. The maternal diet and the hormonal milieu during gestation are claimed up- or down-modulating DNA methylation at global and region levels (Hochberg et al., 2011; Dominguez-Salas et al., 2014). The epigenetic effects of the intrauterine environment affect the phenotype throughout childhood and adulthood (Lee 2015). In an epigenome-wide association study (EGWAS), a specific pattern of CpG methylation in cord blood was significantly associated with birth weight in a large cohort of Norwegians (Engel et al., 2014).

A couple of studies reported that n-3 PUFA supplementation during childhood modulates DNA methylation at this age (Lind et al., 2015; Voisin et al., 2015).

Studies have investigated epigenetic effects of supplementation with selected n-3 PUFAs in pregnancy (Lee et al., 2013; Amarasekera et al., 2014; Lee et al., 2014; Lind et al., 2015; Voisin et al., 2015). No study has investigated the effect of n-3 PUFA deriving exclusively from the maternal diet during pregnancy in absence of supplementation. It is unknown whether the maternal dietary intake of n-3 PUFAs influences shaping of the epigenome during the intrauterine period when it has the greatest plasticity. It is also unclear whether different genomic regions show varying sensitivities to n-3 PUFA content during this vulnerable period.

Aim of the present study was to investigate the association between maternal n-3 PUFA erythrocyte content and DNA methylation profiles of offspring cord blood white cells at birth using whole-genome DNA methylation approaches. The study was designed as “hypothesis generating” investigation to identify candidate genes differently methylated in association with maternal n-3 PUFAs.

## Material and Methods

### Study Design

The primary aim of the “Feeding fetus’ low-grade inflammation and insulin-resistance” cohort study was to investigate the association between maternal intake of lipids during pregnancy and offspring inflammation and insulin resistance at birth (Cinelli et al., 2016). Lipid content of erythrocyte membranes would reflect the maternal dietary fatty acid consumption in the prior 120 days before erythrocyte collection. For a full description of the parent study, see Cinelli et al. (2016). Briefly, healthy pregnant women (N = 1,000) were enrolled at the first trimester and followed up throughout the pregnancy according to guidelines of the Italian Society for Gynecology and Obstetrics (www.sigo.it). Inclusion criteria were ages 18–39 years old, planned pregnancy with folic acid supplementation starting 1 to 3 months before the conception, weeks 7–10 of gestation, on-going folic acid supplementation, singleton pregnancy, no alcohol or medications, no systemic, chronic, or autoimmune disease, no previous diagnosis of gestational diabetes mellitus or miscarriage, no conception through ovulation induction or *in vitro* fertilization, and planned delivery at San Camillo Forlanini Hospital.

During pregnancy, nutritional counseling was provided to all the pregnant women participating to the study (45 min in person meeting with an expert dietician). Pregnant women were encouraged to consume 60g/day of proteins, 45–64% of daily calories from carbohydrates with approximately six servings of whole grain and three servings of fresh fruit daily and 20–35% of daily calories from fats. Three servings per week of fish, particularly salmon, sardines, and anchovies were recommended. Frequencies of food consumption were investigated by a food frequency questionnaire at the study entry and around weeks 13–15 and 23–26.

Pregnant women were also encouraged to exercise regularly (i.e., 45 min walking) (Rasmussen and Yaktine, 2009; Kominiarek and Rajan, 2016).

For the purpose of this ancillary study, we excluded from the cohort of 847 mother–newborn pairs, mothers with history of smoking (N = 93), gestational diabetes mellitus and/or obesity (N = 41), small (N = 49) or large for gestational age (N = 68) newborns, and/or with distress at birth (N = 19, Apgar score at 1 and/or 5 min <7). We calculated quartiles of maternal n-3 PUFAs expressed as ng% in the sample of 577 mothers. We identified the three groups of low n-3 PUFA (N = 144, n-3 PUFA concentration <25th percentile), medium (N = 269, n-3 PUFAs between 25th and 75th percentiles), and high n-3 PUFA content (N = 144, n-3 PUFAs >75th percentile). Then, by using STATA command “sample” (STATA 13.1; StataCorp LP), we randomly drawn, in equal number for male/female, a sample of 39 pairs out of 144 belonging to the low and the high n-3 PUFA groups and of 40 out of 269 from the medium n-3 PUFA group.

The feeding study and the ancillary studies were approved by the Ethical Committee of the Bambino Gesù Children’s Hospital and the San Camillo Forlanini Hospital. The study conformed to guidelines of the European Convention of Human Rights and Biomedicine for Research in Children as revised in 2008. Parents or responsible guardians provided written informed consent. All measures were taken to ensure participants’ confidentiality.

### Newborns’ Anthropometrics

Newborns’ body weight, length, and head circumference were evaluated at birth according to standardized procedures. Standard deviation scores (SDS) for infant weight and height were calculated following the Italian Neonatal Study Chart (Bertino et al., 2010).

### Samples Collection, Lipid Profiling

Maternal blood was withdrawn at fasting, 12–24 h before giving birth, during the pre-partum fetal monitoring. Blood was placed in ethylenediaminetetraacetic acid tubes. Erythrocyte membranes were isolated within 2 h after collection: plasma was separated by centrifugation (980 rpm, 18 min), whereas erythrocytes were added with acid citrate dextrose, washed with distilled water (10:1), and centrifuged (4,000 rpm, 5 min) four times. Erythrocytes were frozen immediately at −80°C and stored until lipid extraction. Briefly, erythrocyte fatty acids were measured by gas chromatography as described previously (Cinelli et al., 2016).

Erythrocyte n-3 PUFAs were expressed as percent weight fraction of the total amount of fatty acids quantified on erythrocyte membranes (Hodson et al., 2008).

### DNA Extraction and Methylation Analysis

Cord blood samples (2.5 ml) were collected at birth by venipuncture from the placental portion of the umbilical cord immediately after clamping. White cells were isolated from cord blood. DNA was extracted using QIAMP Blood Mini Kit (Qiagen, Hilden, Germany). Genomic DNA was extracted, followed by bisulfite conversion (EZ_DNA methylation Kit, Zymo Research, D5002), and methylation analysis (Illumina Infinium Methylation Assay; HumanMethylation450 BeadChip) was used for the epigenome-wide association study performed according to a standardized protocol (http://www.gqinnovationcenter.com/index.aspx) . Arrays were verified for quality control and submitted to the Gene Expression Omnibus. Commercially available custom Methyl Profiler PCR array from Qiagen SABiosciences is used for confirmation of a representative number of hypo- and hypermethylated genes (http://www.sabiosciences.com/dna_methylation_custom_PCRarray.php) using the standard 2-DDCt. Methylation profiles were compared between different samples.

### Glossary

In the text, the terms “probe” or “site” refer to any “CpG site,” a punctual CpG methylation genomic site. CpG is shorthand for 5’-Cytosine-phosphate-Guanine-3’, that is, cytosine and guanine separated by one phosphate group. Cytosines in CpG dinucleotides can be methylated to form 5-methylcytosines. A “region” is a genomic DNA portion, belonging to any of the following categories: “gene body region” (Ensembl genes), “gene promoter region” (promoter regions of Ensembl genes), or “CpG islands” (CpG island track of the UCSC Genome Browser). A CpG island is a region with a high frequency of CpG sites. Hence, we refer to differential mutilated probes/sites (DMPs) and to differentially methylated regions (DMRs), and more specifically to differentially methylated genes (DMGs) as differential methylated gene bodies regions.

### Statistics and Differential Analysis

Descriptive statistics were presented as mean ± SD, median (interquartile range), or frequencies and percentages, as appropriate. Normality of the data distributions was tested by using Kolmogorov–Smirnov’s test with Lilliefors correction.

R packages (“RnBeads,” “methylumi,” “minfi”) were used for the statistical analysis and to explore the chance of a strong differential methylation pattern among the three groups of samples. The background was subtracted using the “noob” method. The signal intensity values were normalized using the SWAN normalization method (Triche et al., 2013), as implemented in the “minfi” R package. In the filtering analysis, 10,119 sites were removed because overlapped with known single nucleotide polymorphisms. Data quality was assessed by using principal component analysis (PCA) and multidimensional scaling (MDS) using both Manhattan and Euclidean distances to identify differentially methylated sites.

Raw methylation level was expressed using the standard beta value (β). β was the estimate of methylation level using the ratio of intensities between methylated and unmethylated alleles. β was between 0 and 1 with 0 being unmethylated and 1 fully methylated.

P-values were calculated using limma t-test and then combined for each region using a generalization of Fischer’s method, the Makambi Weighted inverse chi-squared test (Makambi, 2003). Combined p-value is a good method to avoid multiple comparison issue and increase statistical power. Calculating statistical comparisons on larger genomic regions rather than on simple CpGs allow neighboring CpGs with similar differences in DNA methylation and providing more robust results (Bock, 2012; Müller et al., 2019).

**Figure 1:**
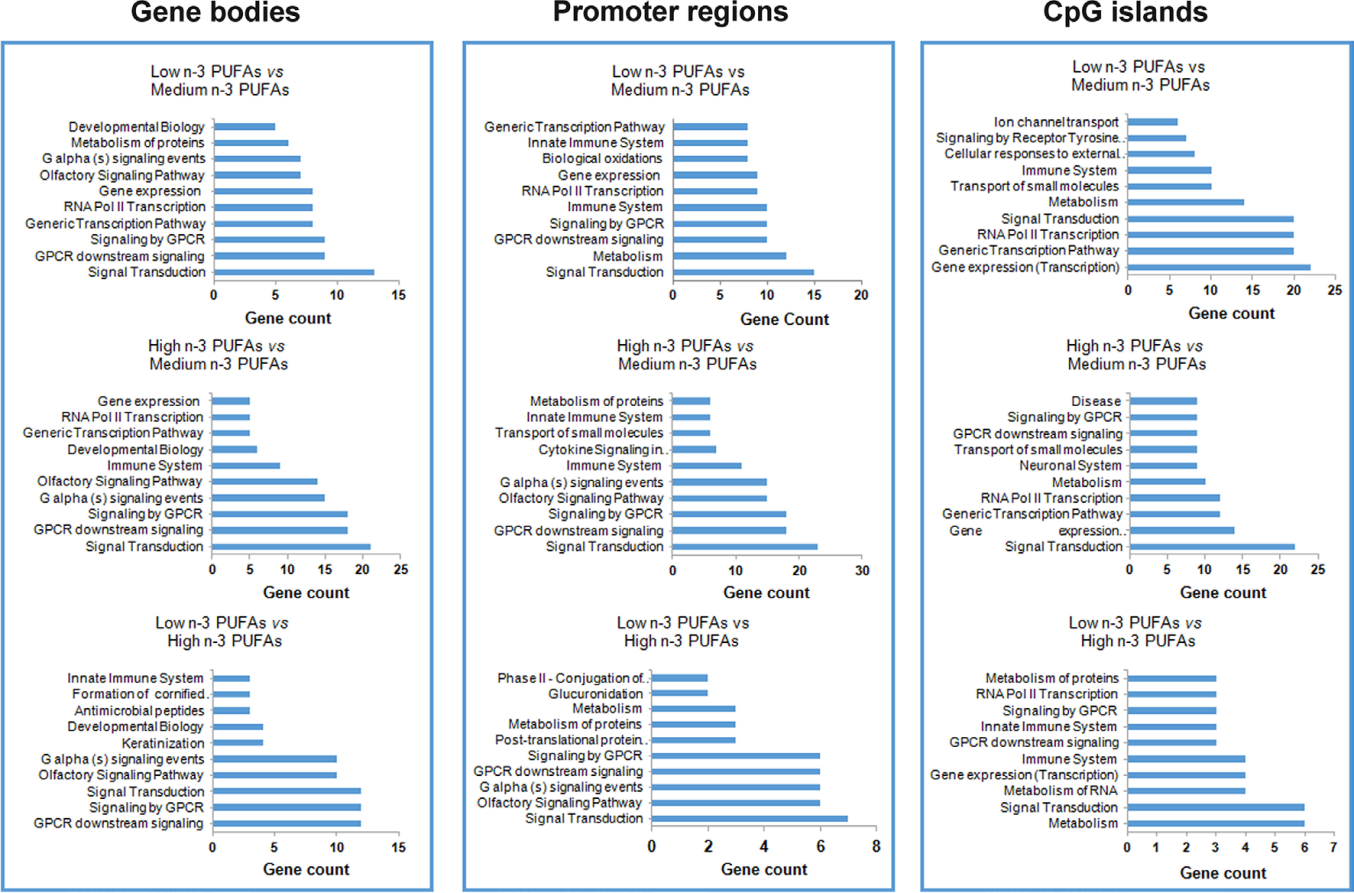
Pathway enrichment resulting from differential methylation profiles associated to n-3 PUFA content. Bar plots of the 10 most abundant pathways resulting from differential methylation profiles at site and region level in low n-3 PUFAs *vs*. medium n-3 PUFAs, high n-3 PUFAs *vs*. medium n-3 PUFAs, and low n-3 PUFAs *vs*. high n-3 PUFAs.

RdBeads R package provides a combined rank number, which it is not intended as a statistical significance test *per se* but can be useful (especially for plots, see **Figure 1**) to have a rapid overall idea of each comparison’s relevance, both from the statistical significance and the magnitude of differential methylation point of view. It is because combined rank is calculated as a combination of the combined p-value, the between group differences of methylation means, and the quotient between group methylation means. The lower the combined rank, the more interesting the comparison is expected to be.

Statistical significance was set at combined P value <0.05. False discovery rate (FDR) statistics was applied to test for type I error in null hypothesis.

### Pathway Enrichment Analysis

Metabolic pathways associated with low or high methylated genes were identified with targeted questions by using Reactome (Fabregat et al., 2016). Reactome pathways over-represented (enriched) in the submitted data derived from the statistical test (hypergeometric distribution) analysis. This test produced a probability score, which was corrected for FDR using the Benjamani–Hochberg method. Venn diagrams showed the overlapping between the overrepresented Reactome pathways and the related different methylated genes obtained (http://bioinformatics.psb.ugent.be).

## Results

### Study Population and Global Differential DNA Methylation Across Groups

One-hundred eighteen newborns were enclosed in the study following the criteria described in the material and method section being born to mothers with low erythrocyte concentration of n-3 PUFAs (concentration <25°percentile), medium n-3 PUFAs (concentration ≥25° percentile and ≤75° percentile), and high n-3 PUFAs (concentration >75° percentile).

Newborns’ anthropometrics and maternal concentrations of n-3PUFAs in the three groups were reported in **Table 1**.

**Table 1:** Anthropometrics of mother–newborn pairs and n-3 PUFAs content (percent of total fatty acids) in maternal erythrocytes.

	Low n-3 PUFAs	Medium n-3 PUFAs	High n-3 PUFAs	*p*
	N = 39	N = 40	N = 39	
**Newborns**				
Gender (M/F)	19/20	20/20	19/20	0.2
Gestational age (weeks)	39.13 ± 1.05	38.8 ± 1.45	39.36 ± 1.16	0.35
Birth weight (g)	3292 ± 284	3357 ± 424	3354 ± 280	0.528
Birth length (cm)	50.2 ± 1.8	50.5 ± 2.2	50.7 ± 1.6	0.448
Birth weight z-score (SDS)	0.024 ± 0.62	0.024 ± 0.84	0.055 ± 0.62	0.529
**Mothers**				
Age (years)	33.0 ± 5.45	33.7 ± 4.8	32.6 ± 5.7	0.853
Pre-pregnancy BMI (kg/m^2^)	21.9 ± 2.9	21.5 ± 2.5	21.8 ± 2.2	0.690
Gestational weight gain (kg)	15.0 ± 5.45	13.9 ± 4.3	12.95 ± 5.2	0.184
Erythrocytes n-3 PUFAs (%)	2.01 ± 1.26	4.34 ± 2.28	6.01 ± 2.34	<0.0001

Methylation analysis revealed no significant difference in the overall methylation profiles of the three groups by using PCA and MDS both at the Manhattan and the Euclidean distances. Next, we performed a differential methylation analysis among groups. We found differential methylations both at site (DMPs) and region levels (DMRs). In **Table 2**, we reported statistically significant DMPs and DMRs at comparison among the three groups (combined p-value ≤ 0.05 for all the between-group comparisons). **Supplementary Figure 1** shows volcano plots (DMR distribution) of DMRs among the three groups, at gene level, promoter regions, and CpG islands. **Supplementary Tables 1**–**3** report data on DMRs in regions (i.e., gene bodies, promoter regions, and CpG islands) at comparison among the three groups. The analysis highlighted a region that was differentially methylated between low and high n-3 PUFA groups. It resulted in the most significant one by combined p-value (p=4.06^06, ∼10^3 times lower than the next best one), and also, it has the best FDR statistics (FDR = 0.1, ∼9 times lower than the next best one). It was a CpG island located on chromosome 7 (start 917633; end 917859) between two genes, COX19 (cytochrome c oxidase assembly factor COX19) and ADAP1 (ArfGAP with dual PH domains 1).

**Table 2:** Number of differential methylations among groups (combined p-value < 0.05).

	Differentially methylated sites	Differentially methylated regions		
		Gene bodies	Promoter regions	CpG islands
Low n-3 PUFAs *vs.* medium n-3 PUFAs	12,716	209	263	220
High n-3 PUFAs *vs.* medium n-3 PUFAs	18,148	317	369	205
Low n-3 PUFAs *vs.* high n-3 PUFAs	8,503	121	131	10,660

### Differentially Methylated Genes and Pathway Enrichment

We identified DMGs in correspondence of DMRs (p < 0.05). They were located in gene bodies (low n-3 PUFAs *vs*. medium n-3 PUFAs, N = 109; high n-3 PUFAs *vs.* medium n-3 PUFAs, N = 130; low n-3 PUFAs *vs.* high n-3 PUFAs N = 59), promoter regions (N = 133; N = 181; N = 61, respectively), and CpG islands (N = 146; N = 135; N = 87) (**Supplementary Tables 4**–**6**). **Supplementary Figure 2** reports Venn diagrams showing number and list of DMGs for each site (gene bodies, promoter regions, and CpG islands) that were low or high methylated in low *vs*. medium and, conversely, high *vs*. medium n-3 PUFA groups. Interestingly, there were DMGs in common between the groups that were methylated either in the same or in the opposite direction.

Then, we performed pathway enrichment analysis of all of these DMGs. As shown in **Figure 1**, DMGs were overrepresented in 10 pathways. Most DMGs in gene bodies belonged to signal transduction—signaling by G-protein coupled receptor (GPCR) and GPCR downstream signaling pathways (**Figure 1A**). Most of DMGs in promoter regions belonged to signal transduction—signaling by GPCR, GPCR downstream signaling, and metabolism and olfactory signaling pathways (**Figure 1B**), and DMGs in CpG island belonged to signal transduction—signaling by GPCR and GPCR downstream signaling pathways (**Figure 1C**).

**Figure 2:**
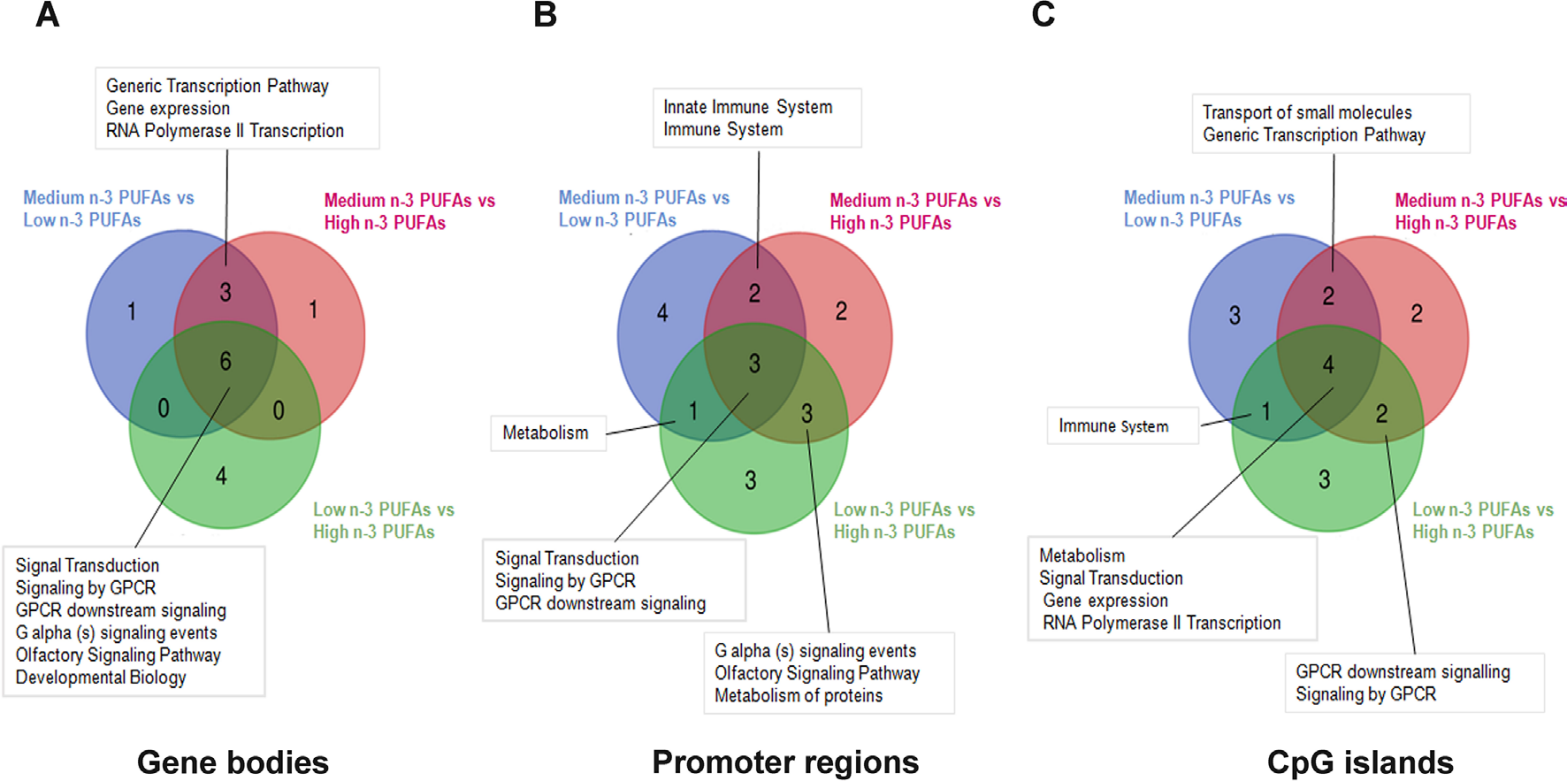
Graphical representation of common inter-group over-represented pathways. Venn diagrams reported the overlapping relationship between the enriched pathways emerging from differential methylation profile at gene bodies **(A)** promoter regions **(B)** and CpG islands **(C)** in the three groups (Low n-3 PUFAs vs. Medium n-3 PUFAs; High n-3 PUFAs vs. Medium n-3 PUFAs; and Low n-3 PUFAs vs. High n-3 PUFAs).

**Figure 2** shows overlap of enriched pathways in gene bodies (**Figure 2A**), promoter regions (**Figure 2B**), and CpG islands (**Figure 2C**) after group comparisons.

**Figure 3:**
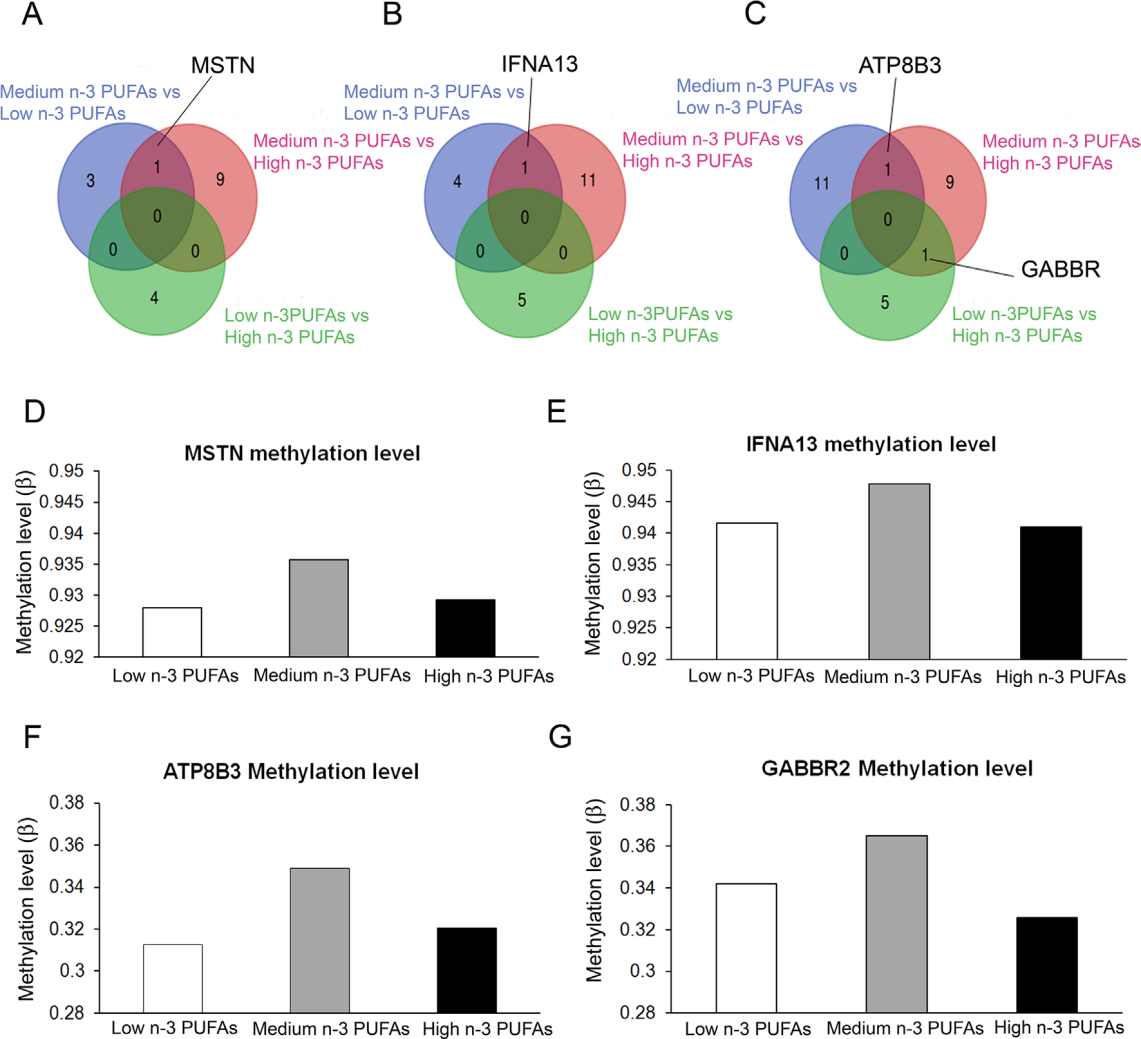
Graphical representation of inter-group common genes. **(A**–**C)** Venn diagrams showing the overlapping of genes with different methylation level associated to the enriched pathways highlighted emerging from differential methylation profile at gene bodies **(A)**, promoter regions **(B)** and CpG islands **(C)** in the three groups (Low n-3 PUFAs vs. Medium n-3 PUFAs; High n-3 PUFAs vs. Medium n-3 PUFAs; and Low n-3 PUFAs vs. High n-3 PUFAs). **(D**–**G)** Histograms representing the methylation level of the four common genes resulting from the analysis among groups.

We found three genes that were differentially methylated in both low and high n-3 PUFA groups as compared to medium n-3 PUFA group (**Figures 3A–C**): the *myostatin* gene (*MSTN*), the *interferon alpha* 13 gene (*IFNA*13), and the *ATPase phospholipid transporting 8B*3 gene (*ATP*8*B*3). The *gamma-aminobutyric acid type B receptor subunit* 2 (*GABBR*2) gene was differentially methylated between medium and high n-3 PUFA groups. Methylation levels of the four genes with different methylation profiles are reported in **Figures 3D–G**.

## Discussion

Findings of the present study support the notion that maternal dietary intake of n-3 PUFAs during pregnancy influences the offspring DNA methylation profile. Differently from previous studies (Lee et al., 2013; Amarasekera et al., 2014; Lee et al., 2014; Lind et al., 2015; Voisin et al., 2015), we explored the association of offspring DNA methylation patterns with content of n-3 PUFAs on maternal erythrocyte membranes in the absence of any n-3 PUFA supplementation. Pregnant women were, indeed, not supplemented but received nutritional counseling.

DNA methylation is an epigenetic mechanism that occurs on the carbon-5 position of cytosines within gene bodies, promoter regions, and, more frequently, CpG islands. DNA methylation induces typically a down-modulation of the gene expression (Manco and Dallapiccola, 2012).

The present study builds upon emerging literature suggesting dietary fats can exert an epigenetic regulation in humans influencing global DNA methylation patterns (Burge and Lillycrop, 2014; Tremblay et al., 2017). In a study of overweight and obese individuals supplemented with 3 g of DHA, 55 gene pathways were significantly overrepresented in supplemented people *versus* controls after 6-week treatment. Sixteen of these pathways were related to inflammatory and immune response, lipid metabolism, type 2 diabetes, and cardiovascular disease (Tremblay et al., 2017). In the Yup’ik Alaska Native people who have a diet rich in n-3 PUFAs from fish oil, the erythrocyte n-3 PUFA content was inversely related to the methylation of *interleukin*-6 (*IL*) gene, thus suggesting that n-3 PUFAs exert anti-inflammatory action also by imposing epigenetic changes (Ma et al., 2016). High n-3 PUFA intake was associated also with 27 other differentially methylated CpG sites at biologically relevant regions, including *FAS*, a key gene of the cell apoptosis (Aslibekyan et al., 2014a).

With regard to n-3 PUFA supplementation in pregnancy, a study of 261 Mexican pregnant women, daily supplemented with 400 mg docosahexaenoic acid (DHA) (N = 131) or placebo (N = 131) from 18 to 22 weeks of gestation to parturition, demonstrated no significant difference in promoter methylation levels of *Th*1, *Th*2, and *Th*17 and regulatory T-relevant genes between n-3 PUFA supplemented and not-supplemented controls. Nevertheless, in infants of mothers who smoked during pregnancy, supplementation was significantly associated with changes of methylation levels in LINE1 repetitive elements and of the promoter methylation levels in *IFN-γ* and *IL*13 genes (Lee et al., 2013). Furthermore, the DHA supplemented group of preterm infants had DNA methylation levels in *IGF*2 *P*3 significantly higher than controls. In addition, at H19 DMR, methylation levels were significantly lower in the DHA group than in controls of normal weight mothers (Lee et al., 2014). In a study of 70 mother–newborn pairs supplemented with 3.7 g of fish oil, association between methylation levels of cord blood CD4+ T with DHA, EPA acid, or total n-3 PUFA levels was suggestive of a dose-dependent effect (Amarasekera et al., 2014).

In the present series, we found DMGs that belonged prevalently to pathways of signal transduction, metabolism, downstream signaling of GPCR, and gene expression among others. Within these pathways, we identified four DMGs, namely, *MSTN*, *IFNA*13, *ATP*8*B*3, and *GABBR*2 that have potential to affect the infant’s metabolic programming. They are related to the onset of insulin resistance and adiposity, to the innate immunity response that is ancestral response to insults including nutrients, to the fatty acid transfer across cellular membranes, and to the reward/addiction paths of the central nervous system (Folmer et al., 2009; Zhang et al., 2011; van der Mark et al., 2013; Aberg et al., 2018; Liu et al, 2018; Martins de Carvalho et al., 2018; Ramos-Lopez et al., 2018). They were low methylated in groups of newborns from mothers with either low or high content of n-3 PUFAs respect to those born to mothers with medium levels. Therefore, we expect these genes overexpressed in both conditions of low and high n-3 PUFA intake.

MSTN is a key protein in the regulation of energy metabolism and muscle insulin resistance. Its overexpression is associated with impaired glucose disposal mostly owing to inhibition of glucose transporter type 4 expression, integration into cytoplasmic membranes, and glucose uptake (Liu et al., 2018). MSTN overexpression leads to indirect suppression of 5’ AMP-activated protein kinase activation and, as such, impacts on the overall energy metabolism (Zhang et al., 2011). Conversely, engineered pigs (Mstn−/−) that lack MSTN present with enhanced insulin sensitivity reduced subcutaneous adipose tissue and increased browning of the latter tissue.

ATP8B3 belongs to the lipid flippases_P4 ATPases that are multispan transmembrane proteins implicated in phospholipid translocation from the exoplasmic to the cytoplasmic leaflet of biological membranes. ATP8B3 is highly expressed in testis, endometrium, spleen, and bone marrow (Folmer et al., 2009 van der Mark et al., 2013). Levels of the protein and its tight regulation by methylation mechanisms may be related to the fine regulation of maternal-fetus fatty acid transfer across the placenta.

GABBR2 seems to be involved in mechanisms of addiction to high fat and alcohol consumption (Martins de Carvalho et al., 2018). Reduced methylation of the gene has been associated with risk of major depression (Aberg et al., 2018) and enhanced sweet taste induced signaling in the 474 adults from the Methyl Epigenome Network Association project (Ramos-Lopez et al., 2018).

IFNA13 is involved in immune-regulatory activities and antiviral response and its relationship with fatty acids metabolism may deserve investigation.

In that, epigenetics may contribute to the fine regulation of fatty acid transfer across the placenta. N-3 PUFAs are essential for the embryo development and the maternal well-being. The placenta modulates the transfer acting as endocrine organ that regulates fetal health growth and maternal metabolism. Fatty acids are major player in the maternal-fetus cross-talk and insufficient or, conversely, excessive amount of these essential fatty acids are detrimental for the fetus’ health (Larqué et al., 2014).

We are aware of several caveats. We do not have genotype or transcription information at the differentially methylated loci, and thus, our understanding of the underlying biologic mechanisms is limited. Differences in methylation levels reached statistical significance at p statistics but not at the FDR statistics probably because of both small-size effect and sample. Furthermore, DNA methylation levels are specific to the type of cell and tissue (Bell et al., 2012). These methylation profiles in cord blood leukocytes might not represent DNA methylation in other tissues even though patterns are globally conserved (Ma et al., 2014; Ronn et al., 2015; Guénard et al., 2016). Finally, annotation of genetic variants has been deemed as inconsistent across databases, incomplete, and subjective toward known genes and pathway analysis that is statistically unpowered (Aslibekyan et al., 2014b).

Strength of the study was measurement of n-3 PUFA content on erythrocyte membranes to estimate maternal intake. The life of a red blood cell is ∼120 d; therefore, this analysis provided a biomarker to measure objectively n-3 PUFA intake in the pregnant women during the last trimester.

Future investigations based on our findings should include replication in independent large populations and functional analyses of the identified genes. As DNA methylation levels relieve functional effects in specific genomic contexts, there is need of functional studies that take into account effect sizes and genomic context.

In conclusion, this study provides evidence that the maternal intake of n-3 PUFAs during pregnancy influences DNA methylation levels in cord blood white cells of newborns. Studies are needed to investigate the clinical relevance and the biological significance of our findings and to inform about recommendable ranges of daily intake of n-3 PUFAs during pregnancy.

## Data Availability Statement

All datasets generated for this study are included in the Article/**Supplementary Files**.

## Ethics Statement

The feeding study and the ancillary studies were approved by the Ethical Committee of the Bambino Gesù children’s Hospital and the San Camillo Forlanini Hospital. The study conformed to guidelines of the European Convention of Human Rights and Biomedicine for Research in Children as revised in 2008. Parents or responsible guardians provided written informed consent. All measures were taken to ensure participants’ confidentiality.

## Author Contributions

MB and AA: data analysis (pathways enrichment) and interpretation and drafting of the manuscript. MF: DNA extraction and epigenetic profiling. CV and FS: cohort enrollment and follow-up. LR: sample stratification and data analysis. RG: bioinformatics analysis (raw methylation data) and contributed significantly to the revision of the entire manuscript. PV: lipid profiling on erythrocytes. MM: research fund acquisition, conception and design of the study, data analysis and interpretation, and drafting.

All the authors revised the manuscript for important intellectual content; approved the manuscript and agree to be accountable for all aspects of the work in ensuring that questions related to the accuracy or integrity of any part of the work have been appropriately investigated and resolved.

## Funding

The Feeding Study was supported by a research grant from the Italian Ministry of Health to Melania Manco (GR-20102304957). The funder had no role in the design and conduct of the study; collection, management, analysis, and interpretation of the data; preparation, review, or approval of the manuscript; and decision to submit the manuscript for publication.

## Conflict of Interest

The authors declare that the research was conducted in the absence of any commercial or financial relationships that could be construed as a potential conflict of interest.
