# Supplementary figures and images for: Maternal Intake of n-3 Polyunsaturated Fatty Acids During Pregnancy Is Associated With Differential Methylation Profiles in Cord Blood White Cells
Source: Front Genet. 2019 Oct 25;10:1050. doi: 10.3389/fgene.2019.01050 (PMC6824245; doi:10.3389/fgene.2019.01050)

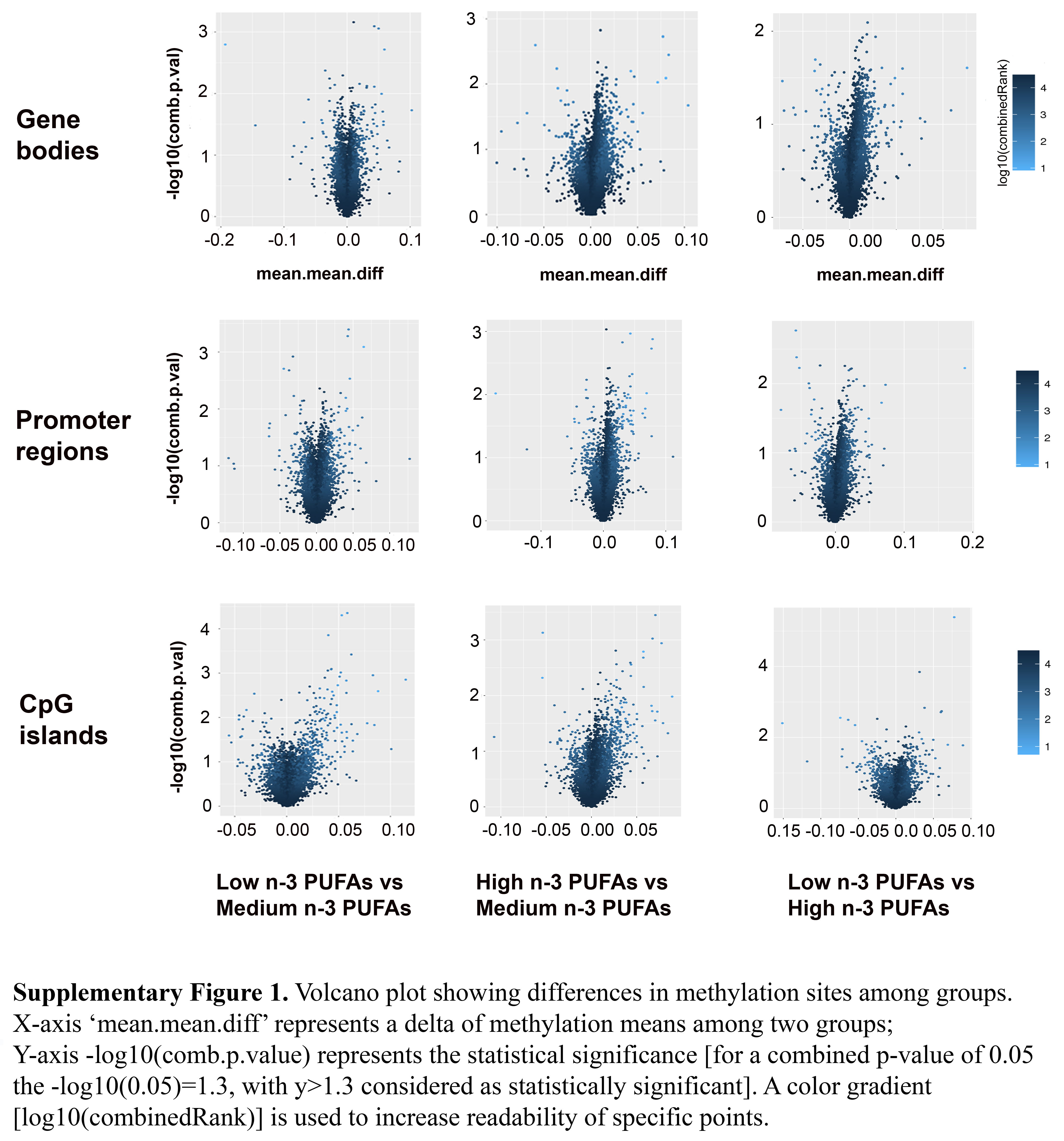

Supplement: Supplementary file 7 [file Image_1.jpeg]

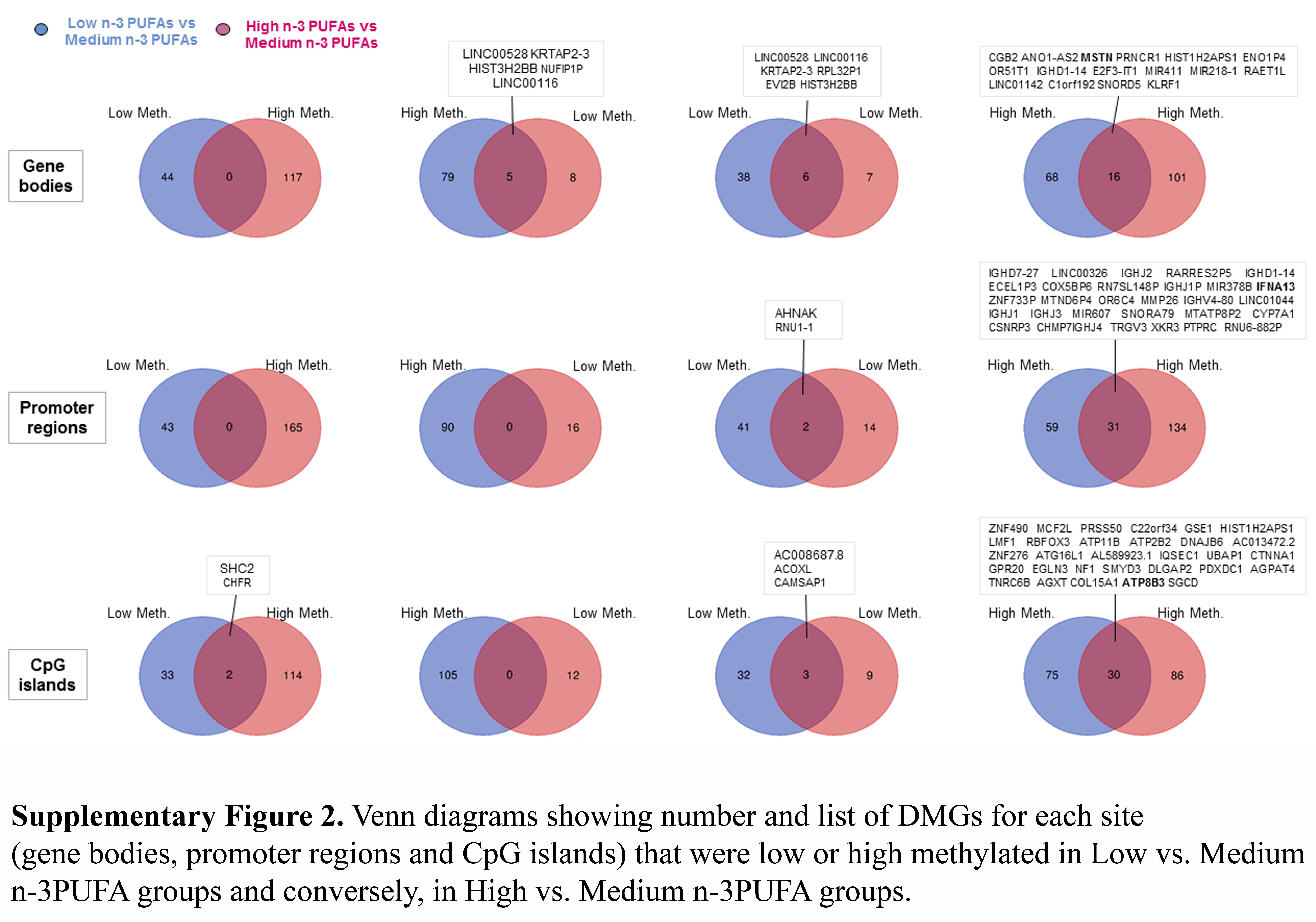

Supplement: Supplementary file 8 [file Image_2.jpeg]
